# A systematic review of patient decision aids for hypertension

**DOI:** 10.1186/s12911-026-03700-0

**Published:** 2026-07-11

**Authors:** Jan Berghold, Lena Fischer, Alexander Pachanov, Leon Vincent Schewe, Dawid Pieper

**Affiliations:** 1 Institute for Health Services and Health System Research, Faculty of Health Sciences Brandenburg, Brandenburg Medical School (Theodor Fontane), Seebad 82/83, Rüdersdorf, 15562 Germany; 2 Center for Health Services Research, Brandenburg Medical School (Theodor Fontane), Rüdersdorf, Germany; 3 SHARE TO CARE. Patient-Centered Care GmbH, Cologne, Germany

**Keywords:** Hypertension, Patient decision aids, Shared decision making, Treatment options, International Patient Decision Aid Standards (IPDAS), Quality assessment, Systematic review

## Abstract

**Background:**

Hypertension is a major cause of premature death and a modifiable risk factor for cardiovascular disease. Effective management includes both pharmacological treatment strategies, including antihypertensive medications, and non-pharmacological treatment strategies, such as lifestyle changes. Shared decision making (SDM) between patients and healthcare professionals is essential to determine the most appropriate treatment for hypertension. Patient decision aids (PDAs) facilitate SDM by providing information on treatment options and helping patients to clarify their values and preferences. However, there is no comprehensive comparison of PDAs for hypertension.

**Objective:**

This systematic review aimed to identify and evaluate PDAs for arterial hypertension using the International Patient Decision Aid Standards (IPDAS) criteria.

**Methods:**

A comprehensive search of bibliographic databases (PubMed, Embase) and gray literature (Google Scholar, Google) was performed in October 2023 and updated in April 2026. Studies and tools were included if they presented a PDA for patients with arterial hypertension, met the IPDAS definition of a PDA and were in German or English. Two researchers screened the literature, extracted data and assessed the quality of the PDAs using the IPDAS Minimal Criteria. Data were synthesized narratively.

**Results:**

Of 1,874 records screened, five PDAs met the inclusion criteria. These PDAs were developed between 2019 and 2024 and originated from the United States, United Kingdom, and Germany. All PDAs addressed the two option areas of medication and lifestyle but differed in the number of individual treatment options presented (range: 2–12 options) and the level of detail in which the options were addressed. All five PDAs were accessible online. Common design features of the PDAs included an option grid format for presenting treatment options with associated benefits and adverse effects, visuals and graphics to enhance comprehension, and value clarification. Quality assessments showed varying levels of compliance with the IPDAS criteria, with overall scores ranging from 49/116 to 87/116.

**Conclusions:**

Few PDAs exist despite the prevalence of hypertension. PDAs varied in quality and format, highlighting gaps in the development process. Further research is needed to evaluate the effectiveness of PDAs for hypertension and to determine which formats are most effective in supporting SDM in different patient populations.

**Clinical trial number:**

Not applicable.

**Supplementary Information:**

The online version contains supplementary material available at 10.1186/s12911-026-03700-0.

## Background

Hypertension, commonly known as high blood pressure, remains a leading cause of premature death and the most significant modifiable risk factor for cardiovascular diseases [1, 2]. According to the 2024 Guidelines of the European Society of Cardiology (ESC) for the management of elevated blood pressure and hypertension [3], arterial hypertension is diagnosed when systolic blood pressure is ≥ 140 mmHg (millimeter mercury) and/or diastolic blood pressure is ≥ 90 mmHg based on office blood pressure measurements. Additionally, if 24-hour ambulatory blood pressure monitoring (ABPM) or home blood pressure monitoring (HBPM) indicates an average systolic blood pressure of ≥ 130 mmHg and/or a diastolic blood pressure of ≥ 80 mmHg, hypertension is also confirmed [3]. Of the nearly 1.3 billion people diagnosed with hypertension worldwide, only about one-fifth have their condition adequately controlled [1]. To reduce the incidence of serious cardiovascular events, such as myocardial infarction, and associated mortality, adequate monitoring and treatment of hypertension is essential [4]. Hypertension therapy comprises two main interventions: pharmacological and non-pharmacological [3]. Pharmacological treatment strategies include the administration of blood pressure-regulating drugs, such as Angiotensin-converting enzyme (ACE) inhibitors or angiotensin receptor blockers (ARBs), calcium channel blockers, thiazide or thiazide-like diuretics, and beta-blockers [3]. Non-pharmacological treatment strategies include lifestyle changes, for example dietary changes, increased physical activity, and weight reduction [1, 3, 5].

With the increasing emphasis on person-centered care and recent research suggesting that a personalized approach to hypertension management can improve adherence to treatment [6], shared decision making (SDM) between healthcare professionals and patients is becoming increasingly important. SDM is a collaborative process in which healthcare professionals and patients work together to make informed medical decisions based on the best available evidence. SDM is advocated as a key strategy to ensure that the choice of treatment is tailored to an individual’s preferences, values and circumstances [6].

Patient decision aids (PDAs) serve as essential tools to support SDM by providing up-to-date, evidence-based information regarding reasonable options and assisting patients clarify their values and preferences related to those choices [7, 8]. For a range of chronic (e.g. cardiovascular disease, diabetes) and acute or interventions-related conditions (e.g. surgery), a recent update of a Cochrane review on PDAs shows that they can help patients make more informed and value-congruent decisions by increasing their knowledge about their condition and encouraging them to take a more active role in decision making [8]. From a behaviour change perspective, PDAs may also support adherence and lifestyle change by increasing patients knowledge and self-efficacy, two factors that feature prominently in established models such as the Theory of Planned Behavior [9]. PDAs are available in a variety of formats, including short or long versions in table or text format, or as web-based versions [10].

Despite increasing policy and guideline support [3] for SDM and in some cases, the use of PDAs in hypertension care and cardiovascular care, their implementation in routine practice remains limited [11, 12]. Previous research suggest that decision about antihypertensive medication are still predominantly clinician-driven and that key components of SDM are present in only a minority of consultations [13]. At the same time, evidence from hypertension and cardiovascular prevention contexts indicates that patients generally view PDAs favorably, reporting improved knowledge, reduced decisional conflict and greater satisfaction with decisions [8]. However, barriers such as time constraints, health literacy demands and limited integration of PDAs into clinical workflows continue to hinder their wider uptake. In hypertension specifically, only a small number of SDM interventions have been evaluated, and effects on SDM measures and blood pressure outcomes have so far been inconsistent [14].

Given the high prevalence of hypertension, the preference-sensitive nature of treatment choices and the variability in how PDAs are developed and reported, a comprehensive comparison is needed to identify available hypertension PDAs, describe their key features and systematically assess their quality. Because the underlying evidence base for antihypertensive treatment is largely shared across countries, differences in PDAs should ideally relate more to design and implementation than to the core treatment options. Currently, there is no comprehensive comparison or quality assessment of existing PDAs for the treatment of arterial hypertension. To address this gap, we conducted a systematic review to identify and evaluate the quality of PDAs for patients with arterial hypertension.

## Methods

In this systematic review of PDAs for arterial hypertension, we followed the Preferred Reporting Items for Systematic Reviews and Meta-Analyses (PRISMA) [15] for reporting, where applicable. The completed PRISMA checklist is provided in Appendix 1. The review was not registered in advance.

### Eligibility criteria

Eligibility criteria were based on the two main elements of the review: (1) PDAs, (2) directed at people diagnosed with arterial hypertension. We employed the International Patient Decision Aid Standards (IPDAS) definition of a PDA, that is: “*tools designed to help people participate in decision making about health care options. They provide information on the options and help patients clarify and communicate the personal value they associate with different features of the options. Patient decision aids do not advise people to choose one option over another*,* nor are they meant to replace practitioner consultation. Instead*,* they prepare patients to make informed*,* values-based decisions with their practitioner”* [7]. This definition is operationalized in the associated IPDAS Minimal checklist [16], i.e. to be included, PDAs had to meet all qualifying criteria of the checklist (see Appendix 4 for qualifying criteria). The population of interest was people with arterial hypertension (office BP: ≥140/90 mmHg; HBPM: ≥135/85 mmHg; daytime ABPM: ≥135/85 mmHg) [3]. The PDAs were included irrespective of care setting or timing of use (before, during, or after consultations), and could be used independently by patients or together with a healthcare professional. We did not impose any age restrictions, but we decided to exclude PDAs for women with pregnancy-induced hypertension. We did not restrict publication dates, but to keep the review feasible, only PDAs in English or German were included.

### Data sources and search strategy

The search was twofold: The database search was designed to identify PDAs published as part of (evaluation) studies, and the gray literature search was designed to identify PDAs not formally published in academic literature.

For the bibliographic database search, one researcher (AP) developed the search strategy for PubMed and adapted it for Embase (Elsevier) (see Appendix 2 for full search strategies). The search was conducted on October 10, 2023 and the PubMed search was updated on April 6, 2026 using the same strategy.

For the gray literature search, we searched Google Scholar and Google in February/March 2024, using a combination of the two key terms “patient decision aid” and “hypertension” – extended by relevant synonyms. We continued to screen Google Scholar and Google pages until we found no relevant web pages/tools or studies for five consecutive pages. An updated web search was conducted in April 2026.

### Study and patient decision aid selection

Two researchers (JB and LF) independently screened all titles and abstracts identified through the bibliographic and gray literature search using Rayyan [17]. After screening the first 50 titles and abstracts, the reviewers met for a discussion session to ensure consistent selection. Potentially relevant full texts were obtained and independently reviewed by the same two researchers. If necessary, a third researcher (DP) was consulted to resolve any disagreements. Reasons for exclusion based on full text were recorded (see Appendix 3). We included all relevant studies that referred to a PDA or, ideally, had the PDA attached/hyperlinked in a list. For relevant studies without directly accessible PDAs (i.e. not linked/attached), we contacted the authors to request the corresponding PDAs. This process resulted in a final list of PDAs, which the two researchers independently checked for eligibility using the a priori defined inclusion criteria.

### Data extraction and analysis

We extracted data into a standardized Microsoft Word table. In addition to general PDA characteristics, including title, author, country, year of development or update, language, number of option areas compared (i.e. medication or lifestyle change), and individual treatment options presented, we extracted the mode of delivery (paper-based or web-based PDA), and PDA design features, such as the method of visual presentation and whether the PDA incorporated a value clarification exercise, a knowledge test, or a risk calculator. Data extraction was carried out by one researcher (JB) and reviewed by another (LF).

### Quality assessment

The IPDAS collaboration provides established criteria and standards to ensure the quality and effectiveness of PDAs [16, 18]. To evaluate the quality of the PDAs included in our review, we applied the IPDAS Minimal Criteria, a 44-item checklist encompassing qualifying, certification and quality criteria [16]. As all qualifying criteria, which can be rated as either present or absent, were required for a PDA to be eligible for inclusion in the review, we already rated them as present at the inclusion stage *(see also eligibility criteria)*. Certification and quality criteria were assessed using a Likert scale ranging from 1 (strongly disagree) to 4 (strongly agree) [16]. The scores assigned for each criterion were subsequently summed to obtain a total score for each PDA. Items for which no information was available in the PDA or related documents were rated as 1 (strongly disagree). Additionally, items that were not relevant to the medical topic of hypertension, such as those related to diagnostic testing, were marked as not applicable (N/A).

One reviewer (JB) assessed the quality of all included PDAs using the 44-item checklist [16], while another (LF) verified the quality assessments. Any discrepancies were resolved through discussion.

## Results

### Search results

**Fig. 1:**
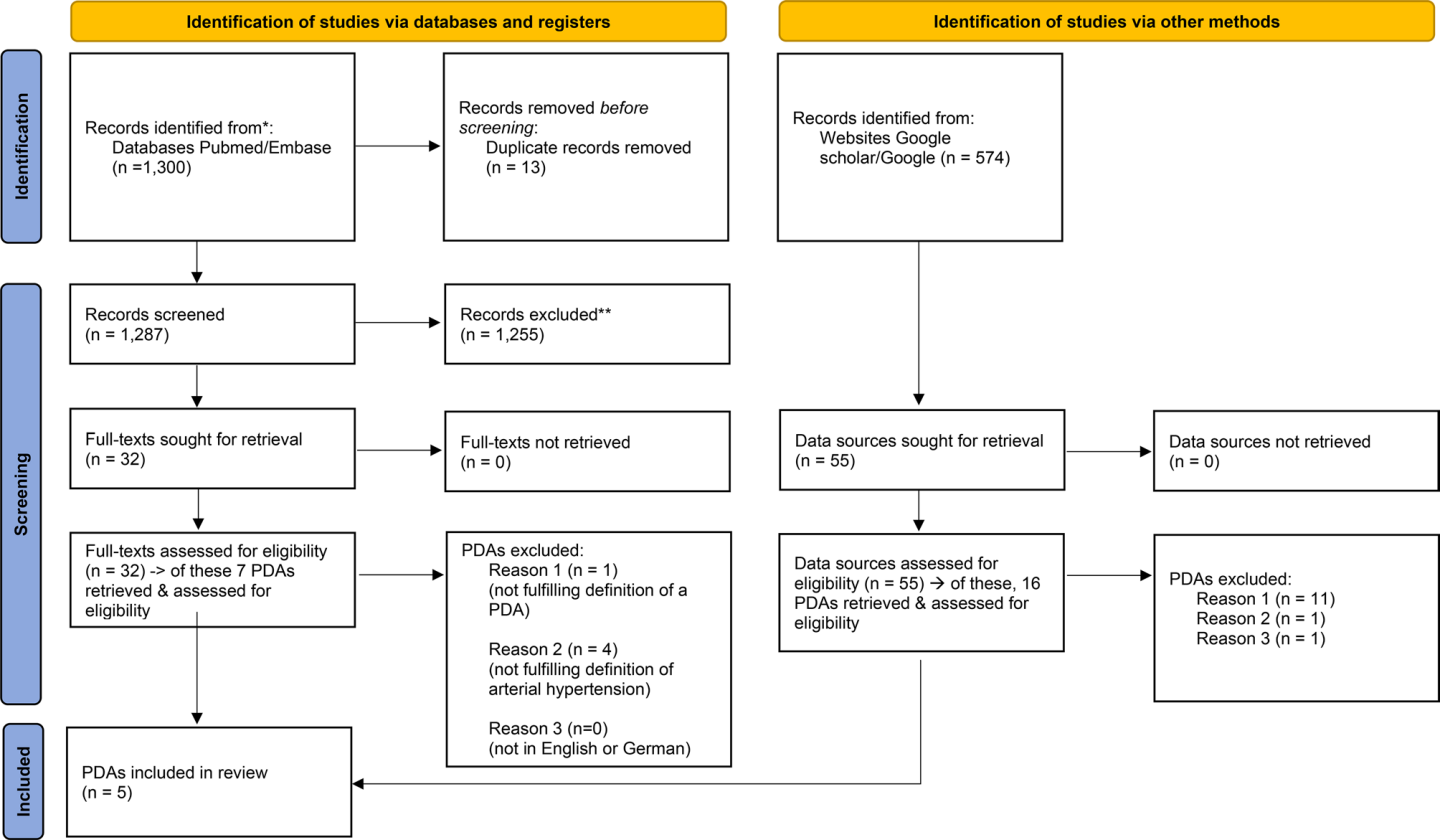
PRISMA 2020 flow diagram for new systematic reviews

We screened a total of 1,874 publications from bibliographic databases and gray literature, from which we identified five PDAs – two from bibliographic databases [19, 20] and three from Google [21–23] – that met the inclusion criteria (Fig. 1). The main reasons for exclusion were that either the studies did not have a PDA attached and we were unable to obtain a PDA after contacting the authors, or the tools could not be classified as PDAs but were purely educational resources or were published in other languages. Additionally, we excluded two decision support tools [24] because they did not meet the IPDAS qualifying criteria (see Appendix 4).

### PDA characteristics

The five PDAs were published by Real General Practice [21], Healthwise [19], SHARE TO CARE [20], the National Institute for Health and Care Excellence (NICE) [22] and the German Institute for Quality and Efficiency in Health Care [Institut für Qualität und Wirtschaftlichkeit im Gesundheitswesen (IQWiG)] [23]. PDA characteristics are shown in Table 1. All were developed or updated within the last five years (i.e. 2019–2023). Two were developed in the United Kingdom (UK) [21, 22], one in the United States [19], and two in Germany [20, 23]. Accordingly, three PDAs were available in English [19, 21, 22], and two in German [20, 23]. All five PDAs were available free of charge [19–23]. Four were directly accessible via a web link [19, 21–23]. For one [20] the Joint Federal Committee provides access data that patients can obtain from their physicians [25]. The mode of delivery of the PDAs included web-based multi-layered formats that users could navigate through step by step [19, 20], an option grid format (i.e. short table-based versions presenting in side-by-side tables the benefits and adverse effects of available treatment options) [21] and a booklet [22, 23].

**Table 1:** Patient decision aid characteristics

ID	Title of the decision aid	Author and/or developing organization	Country; Year (a) developed (b) updated	Language	Target population	Option areas compared	Treatment options presented
1	Blood Pressure Treatment Options https://realgeneralpractice.org/assets /publication/Blood%20pressure% 20PCDA%20January%202,021.pdf	Dr Keith Birrell, Prof James McCormack; Real General Pracitce	UK; (a) 2021 (b) 2021	English	People with high blood pressure; and at risk of heart attacks and strokes	3	**(1) Lifestyle**: (1a) Diet (1b) Exercise (1c) Alcohol (1d) Smoking **(2) Weight loss** **(3) Medication**: (3a) Calcium channel blockers (3b) ACE inhibitors (3c) A2 drugs (3d) Thiazide (3e) Aldosterone antagonists (3f) Beta-blockers (3 g) Alpha blockers
2	High Blood Pressure: Should I Take Medicine? https://www.healthwise.net/ohridecisionaid/ Content/StdDocument.aspx? DOCHWID=zx1768	Healthwise	US; (a) 2023 (b) June 24, 2023	English	People with high blood pressure	2	**(1) Lifestyle** **(2) Medication**
3	High Bloodpressure – How to prevent Cardiovascular Diseases? [Bluthochdruck – Wie Herz-Kreislauf-Erkrankungen vorbeugen? ] https://entscheidungshilfe.share-to-care.de/bluthochdruck/meine-erkrankung/was-bedeutet-meine-erkrankung/	Friedemann Geiger et al.; TAKEPART Media + Science GmbH	Germany; (a) March 31, 2019 (b) -	German	People with high blood pressure	2	**(1) Lifestyle** (1a) Weight (1b) Diet (1c) Alcohol (1d) Smoking (1e) Exercise (1f) Relaxing exercises **(2) Medication** (2a) ACE inhibitors (2b) AT1-receptor antagonists (2c) Calcium channel blockers (2d) Diuretics (2e) Beta blockers
4	How do I control my blood pressure? Lifestyle options and choice of medicines Patient decision aid https://www.nice.org.uk/guidance/ ng136/resources/patient-decision -aid-information-6,899,918,223	National Institute for Health and Care Excellence (NICE)	UK; (a) 2019 (b) 2019	English	People with high blood pressure	3	**(1) Lifestyle** (1a) Diet (1b) Exercise (1c) Weight (1d) Alcohol (1e) Smoking **(2) Medication (+ Lifestyle)** (2a) ACE inhibitor (2b) Angiotensin receptor blocker (2c) Calcium channel blocker (2d) Diuretics **(3) Do nothing**
5	High blood pressure (hypertension): How can I lower it? [Bluthochdruck (Hypertonie): Wie kann ich ihn senken? ] https://www.gesundheitsinformation.de/entscheidungshilfe-bluthochdruck-hypertonie-wie-kann-ich-ihn-senken.html	Institut für Qualität und Wirtschaftlichkeit im Gesundheitswesen (IQWiG)	Germany; (a) December 2023 (b) -	German	People with high blood pressure	2	**(1) Lifestyle** (1a) Weight (1b) Diet (1c) Alcohol (1d) Exercise (1e) Smoking **(2) Medication** (2a) ACE inhibitor (2b) Diuretics (2c) Calcium channel blocker (2d) Angiotensin receptor blocker

*NICE = National Institute for Health and Care Excellence; UK = United Kingdom; U.S = United States

All of the PDAs targeted people diagnosed with hypertension, with one of them specifically focusing on those at risk for heart attack or stroke [21]. The PDAs covered two [19, 20, 23] to three [21, 22] option areas: All included “lifestyle change” and “medication”, with one differentiating between “lifestyle change” and “weight loss” [21] and another explicitly listing the “do nothing” option [22].

The number and level of detail in which option areas or individual treatment options were addressed varied between PDAs (range number of options: 2–12). One PDA [19] broadly compared the option areas “lifestyle” and “medication”, whereas the others provided more detailed comparisons, presenting four to six individual treatment options for lifestyle changes and four to seven different treatment options for the pharmacological treatment of hypertension [20–23]. All PDAs that detailed the option area “lifestyle change” mentioned a healthy diet, regular physical activity/exercise, reduction of alcohol consumption, and smoking cessation. Three mentioned weight loss/maintaining a healthy weight [20, 22, 23], and one mentioned relaxation exercises [16]. For the option area “medication”, four PDAs listed calcium channel blockers, ACE inhibitors, and diuretics [20–23]. Two listed beta blockers [20, 21] and three listed angiotensin II receptor blockers (ARBs), also known as AT1 receptor antagonists [21–23]. One PDA [20] was more specific about the use of AT1 receptor antagonists, which block the angiotensin II type 1 receptor to lower blood pressure. Another included aldosterone antagonists and alpha blockers, which work through different mechanisms to lower blood pressure [21]. The way in which PDAs reported their evidence base differed. One PDA [22] is explicitly referenced in the NICE hypertension guideline [26]. Two PDAs [20, 23] are described as evidence-based and developed by a dedicated evidence team using current German guideline recommendations [27]. A fourth PDA [19] is produced within a structured, URAC-accredited (Utilization Review Accreditation Commission) process in which patient education content is explicitly based on current evidence-based guidelines and regularly updated [28]. The remaining PDA [21] did not explicitly reference guideline documents or primary studies for the treatment options presented.

Table 2 presents detailed information on the content and format of the PDAs. All PDAs provided text-based information specifying the target population and provided condition-related information. Two PDAs [21, 23] provided a link to further information on the diagnosis of high blood pressure (in one PDA the link did not work [21]). All PDAs, either as a main component [21] or as a subcomponent of the whole PDA [19, 20, 22, 23], provided an option grid overview that outlined the reasonable options, along with their benefits, adverse effects and/or impact on daily life. One PDA provided videos [20], three incorporated visuals/graphics [19, 20, 23], two provided personal stories [19, 20], and one presented the absolute risk of experiencing side effects in an icon set [22]. Other features included a knowledge test [19], value clarification exercises (VCE) [19, 20, 23], and links to external websites (e.g. to calculate body mass index) [21]. Two PDAs provided personalized information on outcome probabilities, either through an integrated risk calculator [20] or a reference to one in related documents [22]. One PDA [23] is combined with a web-based patient education tool that provides additional information about aHT in a PDF document linked from the webpage hosting the PDA.

**Table 2:** Patient decision aid features

ID	Mode of delivery/ format	Text	Videos	Visuals/ graphics	Option grid overview	Knowledge test	Value clarification exercise	External link(s)	Personalization of risk/ risk calculator	Patient reports
1	Leaflet/option grid table (options with benefits, harms/side effects)	✓			✓			✓		
2	Multi-layered webbased (facts and key points; option grid overview table; feelings; decision; quiz; summary); print version available	✓			✓	✓	✓			✓
3	Multi-layered webbased; (condition; options with description of benefits/harms/impact to daily life; option grid overview table; my decision); print version available	✓	✓	✓	✓		✓		✓	(✓)*
4	Booklet combining educational information/text and overview tables (options with benefits, harms/side effects); related documents (PDA process and user guide); print version available	✓		✓	✓				✓	
5	Booklet combining educational information/text and overview tables (options with benefits, harms/side effects); value clarification exercise, and link for further information; print version available	✓			✓		✓	✓		

*Patient reports as part of videos

### Quality of the patient decision aids

The detailed quality ratings based on the IPDAS Minimal Criteria checklist [16] are available in Table 3. Of the ten certification criteria, four were not applicable to the topic of hypertension because they pertain to PDAs designed for diagnostic testing. For the six applicable certification criteria, the ratings resulted in combined scores ranging from 13/24 (54%) [21] to 23/24 (96%) [22], with an average score of 19/24 (79%) across the PDAs. Of the 28 quality criteria, five items on diagnostic testing were not applicable. Information about the PDA development process was often missing. One PDA [20] was evaluated in a randomized controlled trial, demonstrating improvements in specific health literacy about hypertension [29]. For the 28 applicable quality criteria, scores ranged from 36/92 (39%) [21] to 68/92 (74%) [20], with an average score of 53/92 (58%) across all five PDAs. When combining scores from both certification and quality criteria, the totals were as follows: Real General Practice 49/116 (42%) [21], Healthwise 65/116 (56%) [19], IQWiG 78/116 (67%) [23], NICE 84/116 (72%) [22], and SHARE TO CARE 87/116 (75%) [20].

**Table 3:** The IPDAS minimal criteria applied to the included patient decision aids for arterial hypertension

No.	Criteria	ID 1 Blood pressure treatment options	ID 2 High Blood Pressure: Should I Take Medicine	ID 3 Bluthochdruck – Wie Herz-Kreislauf-Erkrankungen vorbeugen?	ID 4 How do I control my blood pressure? Lifestyle options and choice of medicines Patient decision aid	ID 5 Bluthochdruck (Hypertonie): Wie kann ich ihn senken?	
Qualifying Criteria (rated: present or absent)							
1	The patient decision aid describes the health condition or problem (treatment, procedure, or investigation) for which the index decision is required.	present	present	present	present		present
2	The patient decision aid explicitly states the decision that needs to be considered (index decision).	present	present	present	present		present
3	The patient decision aid describes the options available for the index decision.	present	present	present	present		present
4	The patient decision aid describes the positive features (benefits or advantages) of each option.	present	present	present	present		present
5	The patient decision aid describes the negative features (harms, side effects, or disadvantages) of each option.	present	present	present	present		present
6	The patient decision aid describes what it is like to experience the consequences of the options (e.g., physical, psychological, social).	present	present	present	present		present
Certification criteria (rated: 1 (strongly disagree) – 4 (strongly agree))							
7	The patient decision aid shows the negative and positive features of options with equal detail (e.g., using similar fonts, sequence, presentation of statistical information).	2/4	4/4	4/4	4/4		4/4
8	The patient decision aid (or associated documentation) provides citations to the evidence selected.	1/4	4/4	3/4	4/4		4/4
9	The patient decision aid (or associated documentation) provides a production or publication date.	4/4	4/4	4/4	4/4		4/4
10	The patient decision aid (or associated documentation) provides information about the update policy.	2/4	2/4	2/4	4/4		4/4
11	The patient decision aid provides information about the levels of uncertainty around event or outcome probabilities (e.g., by giving a range or by using phases such as ‘‘our best estimate is...’’).	3/4	2/4	2/4	4/4		4/4
12	The patient decision aid (or associated documentation) provides information about the funding source used for development.	1/4	3/4	4/4	2/4		3/4
13	The patient decision aid describes what the test is designed to measure.	N/A	N/A	N/A	N/A		N/A
14	If the test detects the condition or problem, the patient decision aid describes the next steps typically taken.	N/A	N/A	N/A	N/A		N/A
15	The patient decision aid describes the next steps if the condition or problem is not detected.	N/A	N/A	N/A	N/A		N/A
16	The patient decision aid has information about the consequences of detecting the condition or disease that would never have caused problems if screening had not been done (lead time bias).	N/A	N/A	N/A	N/A		N/A
Combined score certification criteria		**13/24**	**19/24**	**19/24**	**22/24**		**23/24**
Quality criteria (rated: 1 (strongly disagree) – 4 (strongly agree))							
17	The patient decision aid describes the natural course of the health condition or problem, if no action is taken (when appropriate).	2/4	3/4	3/4	4/4		3/4
18	The patient decision aid makes it possible to compare the positive and negative features of the available options.	2/4	4/4	4/4	4/4		4/4
19	The patient decision aid provides information about outcome probabilities associated with the options (i.e., the likely consequences of decisions).	3/4	1/4	4/4	3/4		3/4
20	The patient decision aid specifies the defined group (reference class) of patients for whom the outcome probabilities apply.	3/4	1/4	4/4	3/4		3/4
21	The patient decision aid specifies the event rates for the outcome probabilities	3/4	1/4	4/4	3/4		3/4
22	The patient decision aid allows the user to compare outcome probabilities across options using the same time period (when feasible).	1/4	1/4	4/4	3/4		3/4
23	The patient decision aid allows the user to compare outcome probabilities across options using the same denominator (when feasible).	3/4	1/4	4/4	4/4		3/4
24	The patient decision aid provides more than 1 way of viewing the probabilities (e.g., words, numbers, and diagrams).	1/4	1/4	4/4	3/4		1/4
25	The patient decision aid asks patients to think about which positive and negative features of the options matter most to them (implicitly or explicitly).	2/4	4/4	4/4	3/4		4/4
26	The patient decision aid provides a step-by- step way to make a decision.	1/4	4/4	4/4	3/4		4/4
27	The patient decision aid includes tools like worksheets or lists of questions to use when discussing options with a practitioner.	1/4	4/4	4/4	3/4		4/4
28	The development process included a needs assessment with clients or patients.	1/4*	1/4*	2/4	3/4		3/4
29	The development process included a needs assessment with health professionals.	1/4*	1/4*	4/4	3/4		3/4
30	The development process included review by clients/patients not involved in producing the decision support intervention.	1/4*	1/4*	1/4*	4/4		1/4*
31	The development process included review by professionals not involved in producing the decision support intervention.	1/4*	4/4	1/4*	4/4		1/4*
32	The patient decision aid was field tested with patients who were facing the decision.	1/4*	1/4*	1/4*	1/4*		1/4*
33	The patient decision aid was field tested with practitioners who counsel patients who face the decision.	1/4*	1/4*	1/4*	1/4*		1/4*
34	The patient decision aid (or associated documentation) describes how research evidence was selected or synthesized.	1/4	3/4	3/4	3/4		3/4
35	The patient decision aid (or associated documentation) describes the quality of the research evidence used.	1/4	2/4	2/4	2/4		2/4
36	The patient decision aid includes authors’/ developers’ credentials or qualifications.	3/4	4/4	4/4	2/4		2/4
37	The patient decision aid (or associated documentation) reports readability levels (using 1 or more of the available scales).	1/4	1/4	1/4	1/4		1/4
38	There is evidence that the patient decision aid improves the match between the preferences of the informed patient and the option that is chosen.	1/4*	1/4*	1/4*	1/4*		1/4*
39	There is evidence that the patient decision aid helps patients improve their knowledge about options’ features.	1/4*	1/4*	4/4	1/4*		1/4*
40	The patient decision aid includes information about the chances of having a true-positive test result.	N/A	N/A	N/A	N/A		N/A
41	The patient decision aid includes information about the chances of having a true-negative test result.	N/A	N/A	N/A	N/A		N/A
42	The patient decision aid includes information about the chances of having a false-positive test result.	N/A	N/A	N/A	N/A		N/A
43	The patient decision aid includes information about the chances of having a false-negative test result.	N/A	N/A	N/A	N/A		N/A
44	The patient decision aid describes the chances the disease is detected with and without the use of the test.	N/A	N/A	N/A	N/A		N/A
Combined score quality criteria		**36/92**	**46/92**	**68/92**	**62/92**		**55/92**
Combined scores certification and quality criteria		**49/116**	**65/116**	**87/116**	**84/116**		**78/116**

*No information or link to related documents for this item can be found in the PDA

## Discussion

### Main findings

Based on this systematic review, we identified five PDAs for arterial hypertension that vary in their mode of delivery, format, and quality. All PDAs address the options areas lifestyle changes and medication, though they differ in addressing individual treatment options and the level of detail in presenting information.

### Findings in context

Compared to other systematic reviews of PDAs, such as those focused on cancer treatment decisions [30] or for screening decisions [31, 32], our review found a limited number of PDAs available for hypertension, despite it being one of the most commonly diagnosed conditions worldwide. The variation in how individual antihypertensive drug classes and lifestyle options are presented across PDAs suggests that approaches to structuring hypertension treatment information differ between tools, even when they address similar guideline-recommended treatment options. One PDA from the US presents only a broad comparison of lifestyle change versus medication without differentiating between individual drug classes, whereas the other PDAs list specific antihypertensive drug classes. Only one PDA explicitly includes „doing nothing“ as an option, which aligns with IPDAS criteria recommending that IPDAs list all relevant option, including the option of not acting or “wait and see”, where appropriate. Final treatment decisions are made through SDM, in which patients preferences are combined with clinical judgement about which antihypertensive drugs are appropriate and safe for the individual patient.

One possible explanation for this may be our language restriction. Other reviews have included PDAs beyond those available in English [30, 31]. However, differences in perceived urgency and irreversibility between cancer and hypertension decisions may also play a role, given that cancer treatment choices are often more definitive, whereas lifestyle changes and antihypertensive medications can usually be adjusted or discontinued over time [33]. In hypertension management, lifestyle changes and medications can be adjusted or discontinued at any time. Our findings are consistent with Hoffmann’s assessment of international PDAs [34], which found significant duplication for certain health areas, while others, including hypertension, remain underserved. This suggests an uneven distribution of resources and highlights the need for targeted PDA development to address chronic, high-prevalence conditions such as hypertension. In addition, structural factors such as limited funding, lack of reimbursement mechanisms and few incentives for clinical implementation may also contribute to the small number of available hypertension PDAs, consistent with reviews highlighting structural barriers to implementing PDAs in routine care [35].

Like other reviews [30–32, 36, 37], the PDAs included in our review vary in meeting quality criteria. Although other reviews used different versions of the IPDAS checklist or excluded some IPDAS criteria, the proportion of IPDAS criteria met in these studies varied, with some reviews reporting compliance as low as 25%, while others found that some PDAs met up to 100% of the criteria were fulfilled in some PDAs [30, 31, 36–38]. This range is consistent with the results of our quality assessment, with 42% − 75% compliance falling within this range. At present, however, there is no consensus on IPDAS cut-off values that would define from which level onward PDAs should be recommended for clinical use, so our scores should be interpreted as descriptive indicators of quality rather than as strict thresholds for implementation. Similar to our review findings, Hoffmann [34] found gaps in development transparency, suggesting a widespread need for clearer documentation in PDA production processes.

Consistent with previous research [39], we found that information about the development process of PDAs was not easy to find or not available at all. This contrasts with the goal of establishing trustworthiness and demonstrating the importance of involving users (i.e. healthcare professionals and patients), for which transparent documentation of the PDA development process is essential [39]. The DEVELOPTOOLS Reporting Checklist [39] provides a comprehensive framework to help PDA developers meet the IPDAS criteria and increase transparency in reporting. As our review focused on the assessment of existing PDAs rather than their development processes, which the checklist is primarily designed to address, it was not a priori relevant to the objectives of our study. However, even if we had wanted to complete the checklist, the PDAs lacked the level of detail required for a thorough application of the DEVELOPTOOLS criteria. Using DEVELOPTOOLS during the initial PDA development process may help to increase reporting quality.

Patient-directed health information should not exceed a 6th to 8th grade reading level to ensure accessibility for a broad population [40, 41]. Although three of the PDAs in our review scored well against the quality standards, we could not find information on readability levels in any of the PDAs. Other studies found that internet-based health information on hypertension vary in quality [42], raising concerns that patients receive misinformation. While high quality PDAs may help to ensure that patients receive information that is correct and understandable, it remains unclear how accessible PDAs are to individuals (with varying levels of (health) literacy) seeking information about hypertension. Future PDA development should therefore routinely include formal readability assessment and transparent reporting of readability levels.

In none of the included PDAs, we found explicit information on whether the PDA should be used individually as a pre or post encounter tool, or together with a healthcare professional. However, the format of four PDAs included in our review suggests that they are primarily intended for pre- or post-encounter use by patients on their own [19, 20, 22, 23]. These PDAs provide some space for notes and/or a print option (e.g. of their VCE) to bring to consultations. The design of the fourth PDA, presented in an option grid format, appears applicable for both pre-/post-encounter as well as during the encounter. While there are various barriers preventing healthcare professionals from recommending and using PDAs with their patients [35], it is currently not known which type of tool (i.e. pre/post-encounter or encounter) is most effective in supporting SDM for diverse groups of patients with hypertension. Recent findings indicate that social determinants of health, including age, race, ethnicity, education and hypertension knowledge, influence the relationship between SDM and blood pressure outcomes [43].

While information on hypertension management can be retrieved through other resources, PDAs can facilitate SDM which may, in turn, improve treatment adherence [6]. In the context of hypertension, where lifestyle changes are central, PDAs may be able to support both the decision making process and behavior change.

### Strengths and limitations of this study

One strength of our systematic review is the comprehensive search strategy, which included both a search in bibliographic databases as well as gray literature. This allowed us to capture PDAs that may be published as part of research articles or available on the internet for practical use. However, due to resource limitations, we did not register our systematic review and only included PDAs in English or German. It is likely that PDAs exist in other languages that would have met our inclusion criteria. In addition, we were unable to update our Embase search because institutional access expired, so very recent studies indexed exclusively in Embase may not have been captured. Additionally, there is an inherent degree of subjectivity in the quality assessment of PDAs, which we attempted to mitigate by having the assessment performed by one researcher, independently reviewed by a second, and then iteratively discussed within the research team. Furthermore, the limited number of included studies and tools precluded meaningful publication bias analyses, which restricts our ability to evaluate whether the available evidence on hypertension PDAs is affected by selective reporting or publication. In addition, the small number of eligible PDAs limits the generalizability of our findings regarding PDA characteristics and quality to the specific tools identified and does not rule out the existence of further hypertension PDAs that are unpublished or not accessible via the sources we searched.

### Implications for research and practice

While we did not specifically look for evidence of formal evaluations of PDAs, none of the included PDAs provided a link to a related evaluation study. However, we did find an evaluation study for the SHARE TO CARE PDA [20]. To address gaps in the evaluation of PDAs, future research is needed to conduct evaluation studies for a variety of endpoints. Aside from the timing of PDA use, it remains unclear which PDA format and features are most useful for patients, particularly when considering different patient groups in terms of risk factors (e.g. smoking) or vulnerability (e.g. health literacy, educational or socioeconomic background). Further research is needed to compare PDAs with different formats and design features.

Our findings also point to several priorities for redesigning and updating hypertension PDAs. Future tools should more transparently document their development process and explicitly link recommendations on lifestyle change and antihypertensive medication to current hypertension guidelines [25, 26]. To better meet the needs of diverse patient groups, hypertension PDAs may benefit from improved readability, clearer visual risk communication and options tailored to different levels of health and digital literacy [8, 14]. In addition, PDAs should be designed for flexible use at different time points along the care pathway (before, during and after consultations) and, where feasible, in digital formats that can be regularly updated and integrated into clinical workflows [8, 44, 45]. Differences in access pathways may also influence real-world use: whereas freely accessible web-based PDAs can, in principle, be found and used directly by patients, tools that are usually accessed via physicians once hypertension has been diagnosed depend more strongly on active recommendation and local implementation in clinical practice. However, for patients with diagnosed hypertension who are already in regular contact with healthcare professionals, physician-mediated access is unlikely to pose a major barrier and may instead help structure the decision process across multiple encounters. In routine care, healthcare providers may therefore prioritise hypertension PDAs that meet recognised quality criteria, reflect current guideline-recommended treatment options, and can be integrated into existing consultation workflows. Equity and inclusion are also important, as hypertension PDAs need to remain usable for patients with limited literacy, restricted digital access and socioeconomic disadvantage.

When trying to assess PDA quality, we noticed that information was often not readily accessible, a challenge that would be even more difficult for patients. More research and practical testing appear needed to find ways to improve transparent reporting of the PDA development process and help PDA developers that reporting can be done with relatively little effort.

Although our review identified high-quality PDAs for hypertension, it remains uncertain how easily patients can find and access these tools. To find out more about PDA accessibility, more research is needed, while a centralized platform that collects PDAs for various conditions would be a valuable resource for both patients and healthcare professionals. Although a single, universally applicable hypertension PDA is unlikely, our findings highlight recurring quality gaps and common design features that can serve as a starting point for more coordinated international efforts in PDA development.

## Conclusions

Although hypertension is one of the most commonly diagnosed conditions worldwide, relatively few PDAs are available to help patients make informed decisions about their treatment. We identified five PDAs, with four of high quality, however, there are gaps in reporting readability levels and transparency in the development process. To further advance PDA development for diverse patient populations, insights are needed on which formats and design features are most effective in supporting SDM. To strengthen practical accessibility, future efforts should also prioritize the development of centralized repositories or platforms that collate PDAs for hypertension and other conditions. Taken together, our review provides practical guidance on what to improve in future hypertension PDAs and which criteria clinicians can use when selecting tools for routine care.

## Supplementary Information

Below is the link to the electronic supplementary material.

Supplementary Material 1

Supplementary Material 2

Supplementary Material 3

Supplementary Material 4

## Data Availability

No datasets were generated or analysed during the current study.

## Abbreviations

ABPM: Ambulatory blood pressure monitoring
ACE: Angiotensin-converting enzyme
ARBs: Angiotensin receptor blockers
BP: Blood pressure
ESC: European Society of Cardiology
HBPM: Home blood pressure monitoring
IPDAS: International Patient Decision Aid Standards
N/A: Not applicable
NICE: National Institute for Health and Care Excellence
PDA: Patient decision aid
PRISMA: Preferred reporting Items for Systematic Reviews and Meta-Analyses
SDM: Shared decision making
UK: United Kingdom
VCE: Value clarification exercises
