# Supplementary material for: A systematic review of patient decision aids for hypertension
Source: BMC Med Inform Decis Mak. 2026 Jul 11;26:259. doi: 10.1186/s12911-026-03700-0 (PMC13355325; doi:10.1186/s12911-026-03700-0)
Supplement: Supplementary file 2 — Supplementary Material 2 [file 12911_2026_3700_MOESM2_ESM.docx]

**Appendix 2.** Search strategies

**PubMed**

Full search strategy:

(("decision aid"[Title/Abstract] OR "decision aids"[Title/Abstract] OR "informed decision"[Title] OR "informed decisions"[Title] OR "informed choice"[Title] OR "treatment decision"[Title] OR "treatment decisions"[Title] OR "treatment choice"[Title] OR "decision support techniques"[MeSH Terms]) AND ("hypertension"[Title] OR "hypertensive"[Title] OR "elevated blood pressure"[Title] OR "elevation blood pressure"[Title:~2] OR "high blood pressure"[Title] OR "increased blood pressure"[Title] OR "increase blood pressure"[Title:~2] OR "hypertension"[MeSH Terms])) NOT ("case reports"[Publication Type] OR "letter"[Publication Type] OR "editorial"[Publication Type] OR "comment"[Publication Type] OR "news"[Publication Type] OR "preprint"[Publication Type] OR "historical article"[Publication Type] OR "interview"[Publication Type])

Search conducted on October 10, 2023 and updated on April 6, 2026 using the same search strategy.

Restrictions: No time period restrictions were applied. Only studies written in English and/or German were included.

**Embase**

Full search strategy:

('decision aid':ti,ab OR 'decision aids':ti,ab OR 'informed decision':ti OR 'informed decisions':ti OR 'informed choice':ti OR 'treatment decision':ti OR 'treatment decisions':ti OR 'treatment choice':ti OR 'decision support system'/exp) AND ('hypertension':ti OR 'hypertensive':ti OR 'elevated blood pressure':ti OR 'elevation near/2 blood pressure':ti OR 'high blood pressure':ti OR 'increased blood pressure':ti OR 'increase near/2 blood pressure':ti OR 'hypertension'/exp) AND [embase]/lim NOT [medline]/lim NOT [pubmed-not-medline]/lim NOT ('case reports':pt OR 'letter':pt OR 'editorial':pt OR 'comment':pt OR 'news':pt OR 'preprint':pt OR 'historical article':pt OR 'interview':pt)

Search conducted on October 10, 2023

Restrictions: No time period restrictions were applied. Only studies written in English and/or German were included.
