# Supplementary material for: A systematic review of patient decision aids for hypertension
Source: BMC Med Inform Decis Mak. 2026 Jul 11;26:259. doi: 10.1186/s12911-026-03700-0 (PMC13355325; doi:10.1186/s12911-026-03700-0)
Supplement: Supplementary file 3 — Supplementary Material 3 [file 12911_2026_3700_MOESM3_ESM.docx]

**Appendix 4.** Qualifying criteria of the IPDAS Minimal criteria checklist

| No. | Qualifying Criteria |
| --- | --- |
| 1 | The patient decision aid describes the health condition or problem (treatment, procedure, or investigation) for which the index decision is required. |
| 2 | The patient decision aid explicitly states the decision that needs to be considered (index decision). |
| 3 | The patient decision aid describes the options available for the index decision. |
| 4 | The patient decision aid describes the positive features (benefits or advantages) of each option. |
| 5 | The patient decision aid describes the negative features (harms, side effects, or disadvantages) of each option. |
| 6 | The patient decision aid describes what it is like to experience the consequences of the options (e.g., physical, psychological, social). |
