# Supplementary material for: A systematic review of patient decision aids for hypertension
Source: BMC Med Inform Decis Mak. 2026 Jul 11;26:259. doi: 10.1186/s12911-026-03700-0 (PMC13355325; doi:10.1186/s12911-026-03700-0)
Supplement: Supplementary file 4 — Supplementary Material 4 [file 12911_2026_3700_MOESM4_ESM.docx]

**Appendix 3.** Reasons for exclusion

| Criterion | Reason for exclusion |
| --- | --- |
| E1 | Does not fulfill the definition of a PDA according to IPDAS. |
| E2 | Does not apply to the decision to treat arterial hypertension according to the definition of the European Society of Cardiology and the European Society of Hypertension. |
| E3 | Does not apply to the target group included. |
| E4 | The PDA is published in another language than English or German. |

**3.1.** Excluded studies - bibliographic database search (PubMed, Embase)

| Nr. | Reference | Reason for exclusion |
| --- | --- | --- |
| 1. | Buhse S, Kuniss N, Liethmann K, et al. Informed shared decision-making programme for patients with type 2 diabetes in primary care: cluster randomised controlled trial. BMJ Open 2018;**8**:e024004. doi:10.1136/ bmjopen-2018-024004 | E2 |
| 2. | Liina Kask-Flight, Koray Durak, Kadri Suija, Anneli Rätsep, Ruth Kalda. Reduction of cardiovascular risk factors among young men wirh hypertension using an interactive decision aid: cluster-randomized trial. BMC Cardiocascular Disorders 2021; 21:543. doi: 10.1186/s12872‐021‐02339‐1 | E2 |
| 3 | Kim DH, Shi SM, Carroll D, Najafzadeh M, Wei LJ. Restricted mean survival time versus conventional measures for treatment decision-making. J Am Geriatr Soc. 2021 Aug;69(8):2282-2289. doi: 10.1111/jgs.17195. Epub 2021 Apr 26. PMID: 33901300; PMCID: PMC8373742. | E1 |
| 3. | Lyne Lalonde, Annette M. O’Connor, Pierrette Duguay, Joëlle Brassard, Elizabeth Drake, Steven A. Grover. Evaluation of a decision aid and a personal risk profile in community pharmacy for patients considering options to improve cardiovascular health: the OPTIONS pilot study. International Journal of Pharmacy Practice 2006; 14: 51-62. doi: 10.1211/ijpp.14.1.0007 | E2 |
| 4. | Taksler GB, Hu B, DeGrandis F Jr, Montori VM, Fagerlin A, Nagykaldi Z, Rothberg MB. Effect of Individualized Preventive Care Recommendations vs Usual Care on Patient Interest and Use of Recommendations: A Pilot Randomized Clinical Trial. JAMA Netw Open. 2021 Nov 1;4(11):e2131455. doi: 10.1001/jamanetworkopen.2021.31455. PMID: 34726747; PMCID: PMC8564576. | E2 |

**3.2.** Excluded studies - gray literature search (Google, Google Scholar)

| 1. | Lalonde L, O'Connor AM, Drake E, Duguay P, Lowensteyn I, Grover SA. Development and preliminary testing of a patient decision aid to assist pharmaceutical care in the prevention of cardiovascular disease. Pharmacotherapy. 2004 Jul;24(7):909-22. doi: 10.1592/phco.24.9.909.36104. PMID: 15303454. | E2 |
| --- | --- | --- |
| 2. | Houston TK, Allison JJ, Sussman M, Horn W, Holt CL, Trobaugh J, Salas M, Pisu M, Cuffee YL, Larkin D, Person SD, Barton B, Kiefe CI, Hullett S. Culturally appropriate storytelling to improve blood pressure: a randomized trial. Ann Intern Med. 2011 Jan 18;154(2):77-84. doi: 10.7326/0003-4819-154-2-201101180-00004. Erratum in: Ann Intern Med. 2011 May 17;154(10):708. PMID: 21242364. | E1 |
| 3. | McCartney M, Treadwell J, Maskrey N, Lehman R. Making evidence based medicine work for individual patients. BMJ. 2016 May 16;353:i2452. doi: 10.1136/bmj.i2452. PMID: 27185764. | E1 |
| 4. | Bonner C, Patel P, Fajardo MA, Zhuang R, Trevena L. Online decision aids for primary cardiovascular disease prevention: systematic search, evaluation of quality and suitability for low health literacy patients. BMJ Open. 2019 Mar 13;9(3):e025173. doi: 10.1136/bmjopen-2018-025173. PMID: 30872547; PMCID: PMC6429890. | E1 |
| 5. | Bonner C, Patel P, Fajardo MA, Zhuang R, Trevena L. Online decision aids for primary cardiovascular disease prevention: systematic search, evaluation of quality and suitability for low health literacy patients. BMJ Open. 2019 Mar 13;9(3):e025173. doi: 10.1136/bmjopen-2018-025173. PMID: 30872547; PMCID: PMC6429890. | E1 |
| 6. | Islip Medical Practice. https://islipmedicalpractice.org.uk/conditions/high-blood-pressure-hypertension/; High blood pressure (hypertension); 27.02.24 | E1 |
| 7. | Real General Practice. https://realgeneralpractice.org/assets/audio/Options%20-%20Douglas%20-%20high%20blood%20pressure%20and%20chronic%20kidney%20disease.%20Options%20to%20reduce%20cardiovascular%20risk%20-%20MQ.mp3; Options to reduce cardiovascular risk when the risk is high; 27.02.24 | E1 |
| 8. | British Heart Foundation. https://www.bhf.org.uk/informationsupport/risk-factors/high-blood-pressure; High blood pressure; 27.02.24 | E1 |
| 9. | Hypertension Canada. https://hypertension.ca/public; Hypertension = High Blood Pressure; 27.02.24 | E1 |
| 10. | Blood Pressure UK. https://www.bloodpressureuk.org/media/bpuk/docs/IntroducingHighBP_Web-(3).pdf; Introducing High Blood Pressure; 27.02.24 | E1 |
| 11. | American College of Cardiology. https://tools.acc.org/ascvd-risk-estimator-plus/#!/calculate/estimate/; ASCVD Risk Estimator Plus; 27.02.24 | E1 |
| 12 | PEER Simplified Lipid Guideline Group. https://decisionaid.ca/cvd/; PEER Simplified Cardiovascular Decision Aid; 27.02.24 | E1 |
